# Characterizing antimicrobial resistance and plasmidome diversity in *Escherichia coli* from imported frozen broiler chicken in the United Arab Emirates

**DOI:** 10.3389/fmicb.2025.1590906

**Published:** 2025-06-16

**Authors:** Ihab Habib, Mohamed-Yousif Ibrahim Mohamed, Glindya Bhagya Lakshmi, Akela Ghazawi, Mushtaq Khan, Hazim O. Khalifa

**Affiliations:** ^1^Department of Veterinary Medicine, College of Agriculture and Veterinary Medicine, United Arab Emirates University, Al Ain, United Arab Emirates; ^2^ASPIRE Research Institute for Food Security in the Drylands (ARIFSID), United Arab Emirates University, Al Ain, United Arab Emirates; ^3^Department of Medical Microbiology and Immunology, College of Medicine and Health Sciences, United Arab Emirates University, Al Ain, United Arab Emirates; ^4^Zayed Center for Health Sciences, United Arab Emirates University, Al Ain, United Arab Emirates

**Keywords:** *Escherichia coli*, antibiotic resistance, imported poultry, whole-genome sequencing, United Arab Emirates

## Abstract

**Introduction:**

The global increase in antimicrobial-resistant (AMR) *Escherichia coli* in the poultry supply chain poses significant food safety and public health risks. This study aims to assess the AMR profiles and plasmid content of *E. coli* isolated from imported frozen broiler carcasses available in the United Arab Emirates (UAE) market.

**Methods:**

A total of 253 frozen whole broiler carcasses imported from Brazil, France, Oman, and Ukraine were screened for the presence of *E. coli*. Antimicrobial susceptibility testing was conducted on 90 isolates. Whole-genome sequencing (WGS) was performed on 33 representative isolates to analyze sequence types (STs), resistance genes, and plasmid content using the MOB-suite pipeline.

**Results:**

*E. coli* was detected in 248 out of 253 samples. Resistance to ampicillin (52.2%) and tetracycline (35.6%) was most common, with 68.9% of isolates exhibiting multidrug resistance (MDR). WGS revealed 22 STs, with ST1564 being the most prevalent (12.1%). Clinically relevant ST10 and ST58 were also identified. Extended-spectrum β-lactamase (ESBL) genes *bla*_*CTX*–*M*–55_ and *bla*_*CTX*–*M*–8_ predominated, often co-occurring with fluoroquinolone resistance genes qnrS1 and qnrB19. A total of 197 plasmids were identified; 63.6% were classified as conjugative. The most frequent relaxase types were MOBP (37 plasmids) and MOBF (24 plasmids), with IncI-gamma/K1 and IncF plasmids commonly linked to ESBL genes.

**Discussion:**

This study provides one of the first genomic characterizations of plasmid-mediated AMR in poultry-associated *E. coli* in the Middle East. The high prevalence of MDR and mobile resistance elements underscores the role of international poultry trade in AMR dissemination. These findings highlight the need for strengthened AMR surveillance and improved regulatory control over antibiotic use in poultry production to mitigate public health risks.

## 1 Introduction

The emergence of foodborne antimicrobial resistance (AMR) poses an important threat to food safety worldwide. Among the vast array of antimicrobial-resistant microorganisms, *Escherichia coli* plays a particularly significant role due to its prevalence in human and animal microbiota (Pitout and Laupland, 2008; Ramos et al., 2020). The high genetic adaptability and ability of *E. coli* to exchange genetic material make them key contributors to the spread of genes conferring antimicrobial resistance (Ibekwe et al., 2021). In poultry and poultry products, multi-drug resistant *E. coli* strains, including the extended-spectrum β-lactams (ESBL), present a pressing One Health challenge, giving the potential of human exposure through food handling and consumption (Ramos et al., 2020). As poultry products, mainly chicken meat, remain one of the most popular internationally traded proteins of animal sources, the global dissemination of such resistant bacteria is highly feasible (Warren et al., 2008).

A key element of surveillance in antimicrobial resistance research involves a comprehensive genetic analysis of the bacteria of interest (Sanderson et al., 2023). A significant number of genes facilitating virulence and antibiotic resistance in *E. coli* are typically located on plasmids (Pitout and Laupland, 2008). Three plasmid types are classified based on their potential to transfer and conjugate: non-mobilizable, mobilizable, and conjugative. Conjugative ones are capable of self-transfer, whereas mobilizable plasmids, which lack some conjugation elements, require the presence of a helper conjugative plasmid for transmission. In contrast, non-mobilizable plasmids cannot transfer between bacteria (Neffe et al., 2022). Various bioinformatics platforms can predict the presence and mobility of plasmids from microbial genome data (Robertson and Nash, 2018). Accurately identifying and characterizing *E. coli* plasmids is essential for understanding their epidemiological impact and public health significance (Sanderson et al., 2023).

The United Arab Emirates (UAE) strategic position as a global trade and tourism hub underscores the risk of receiving and disseminating multi-drug-resistant organisms through internationally traded foods (Habib et al., 2021). Consequently, lapses in screening imported poultry for antimicrobial-resistant pathogens may promote the spread of these strains within and beyond the local market. In the UAE, previous studies have explored antimicrobial resistance among *E. coli* from locally produced fresh/chilled broiler meat, highlighting the high prevalence (79.68%) of ESBL strains (Habib et al., 2023b). Nevertheless, no studies specifically focused on AMR profiling of *E. coli* in imported frozen broiler chicken within the UAE, where about 85% of the volumes of broiler chicken are imported to meet consumer demand (USDA, 2021).

This study addresses existing knowledge gaps by establishing the first baseline on antimicrobial resistance in a subset of generic *E. coli* isolated from broiler meat imported from different countries and presented in the UAE market. Advanced genome sequencing techniques were employed for a subset of 33 isolates to assess genotypic diversity, identify antimicrobial resistance genes, and analyze plasmid mobility and variability, particularly among ESBL-resistant strains. These findings will serve as valuable data for both local and international stakeholders, supporting the integration of genomic tools as novel methodologies in future food safety risk assessments.

## 2 Materials and methods

### 2.1 Samples and isolation of *Escherichia coli*

The sample size was statistically calculated using a binomial proportion-based formula for prevalence studies, assuming an expected *E. coli* detection rate of 75%, a 90% confidence level, and a 5% margin of error, resulting in a minimum required sample size of 201 (Dohoo et al., 2010). To ensure comprehensive coverage and account for diversity in sample sources, 253 samples of whole chicken carcass were collected frozen from the major markets in Al-Ain and Abu Dhabi, UAE, between January and August 2023. The samples originated from four primary exporting countries and were distributed as 144 samples from Brazil (7 brands), 38 samples from France (1 brand), 28 samples from Oman (2 brands), and 28 samples from Ukraine (1 brand). Each sample was labeled, individually packed in polyethylene bags, and transferred in a cooling box to the testing Laboratory at UAE University. Samples were defrosted at 4°C and tested within the following day of collection.

Following defrosting, each chicken carcass was individually transferred to a sterile stomacher bag, weighed, and rinsed with 1% buffered peptone water (BPW; 400 ml) of (Stearns et al., 2024). The carcass was then manually shaken within the sealed bag by grasping both ends of the bag and vigorously agitating it in alternating back-and-forth and up-and-down motions for one minute to ensure thorough dislodgement and collection of surface-associated microorganisms (Stearns et al., 2024). To detect the presence of *E. coli*, 30 mL of the rinsate was added to 270 mL of BPW (1:10 ratio), vortexed, and incubated at 37°C ± 1°C for 24 h (Brichta-Harhay et al., 2007). A 10-μL of the enriched broth was then spread onto Tryptone Bile X-Glucuronide agar (TBX), followed by incubation at 44°C ± 1°C for 24 h. Presumptive *E. coli* colonies were subsequently subcultured on nutrient agar, and after overnight incubation, isolates were characterized using MALDI-TOF MS with the platform Autobio-MS-1000 (Autobio Diagnostics, China) (Habib et al., 2023a).

### 2.2 Testing for antimicrobial susceptibility

A random subset of 90 *E. coli* isolates (approximately 36.3% of the total recovered isolates) was selected for phenotypic antimicrobial susceptibility testing to ensure representative coverage across different sample sources, sampling months, and locations. The susceptibility of the isolates (*n* = 90) was assessed using the method of disc diffusion on Mueller-Hinton agar, following the Clinical and Laboratory Standards Institute (CLSI) guide (CLSI, 2020). A panel of 12 antimicrobials: ampicillin (10 μg), azithromycin (15 μg), ciprofloxacin (5 μg), chloramphenicol (30 μg), gentamicin (10 μg), tetracycline (30 μg), trimethoprim-sulfamethoxazole (25 μg), cefotaxime (30 μg), cefoxitin (30 μg), cefepime (30 μg), ceftriaxone (30 μg), and imipenem (10 μg), was used (Habib et al., 2023b). Isolates were denoted as multidrug-resistant (MDR) if they resisted at least one antimicrobial in ≥ 3 classes based on CLSI-defined breakpoints (Magiorakos et al., 2012).

### 2.3 Isolates for whole-genome sequencing

A subset of 33 isolates was subjected to WGS, in order to provide initial inferences about prevalent resistance genes, sequence types, and phylogenetic patterns. The isolates were carefully chosen to ensure representativeness across different sources, sampling months, and AMR profiles, thereby providing an “initial” picture of the diversity within the population under study. This approach aligns with the recommendations of the European Food Safety Authority (EFSA), which state that for epidemiological surveillance using WGS, it is not necessary to sequence all isolates but rather to ensure that the selected subset is representative in terms of source, geography, time, and phenotypic traits (EFSA Panel on Biological Hazards (Biohaz), 2021). The inclusion strategy adopted in this study provides insightful coverage to investigate the genomic characteristics and AMR determinants of *E. coli* isolates present in the tested food samples. DNA extraction utilized the Wizard^®^ DNA Kit (Promega, United States), followed by a quality check (Ghazawi et al., 2024). Short-read sequencing was done using NovaSeq platform by Novogene (UK).

Raw sequencing reads were quality-checked using FastQC v0.11.9 (Andrews, 2010), trimmed with fastp v0.23.2 (Chen et al., 2018), and *de novo* assembled with Shovill v1.1.0 (Seemann, 2023). Analysis of the genomes was performed using a cloud-based platform (Solu Healthcare, Inc., Finland; Saratto et al., 2025).^1^ Various tools were integrated into the platform, including BactInspector for species identification and multi-locus sequence typing (MLST). *In silico* serotyping of *E. coli* isolates was performed using SerotypeFinder v2.0 (Joensen et al., 2015), available through the Center for Genomic Epidemiology (CGE) platform. Genes conferring AMR were annotated using AMRFinderPlus, with a gene identification threshold of 90% (Feldgarden et al., 2021; Saratto et al., 2025). The default value AMRFinderPlus uses for the minimum coverage is 50% (Feldgarden et al., 2021), but in our sequenced isolates the actual coverage in most cases was greater than 90% [available in Supplementary File (Spreadsheet) S1 (Supplementary Table 1.1)]. A phylogenetic tree was constructed through the Solu platform based on distances of whole-genome single-nucleotide polymorphism (SNP), defining isolates as closely related if they shared ≤ 20 SNPs (Hasan et al., 2021). All raw data of the genome sequencing are publicly available in the National Center for Biotechnology Information BioProject number PRJNA1219370.^2^ Assembly quality metrics of the 33 whole-genome sequenced *E. coli* from frozen imported retail chicken in the UAE are provided in Supplementary File (Spreadsheet) S2.

### 2.4 Predicted plasmid mobility analysis

Plasmid mobility was predicted using the MOB suite platform (v3.1.9) (Robertson and Nash, 2018). The identified plasmid scaffolds are compared against a mobility clusters database (MOB-clusters) to identify their closest match. Putative plasmids were categorized into MOB-clusters and assigned mobility classifications (“Conjugative,” “Mobilizable,” or “Non-mobilizable”) and relaxase gene clusters. The relaxase gene clusters determine the specificity and efficiency of plasmid transfer by recognizing and cleaving the origin of transfer (*oriT*) sites, thereby facilitating the horizontal transfer of antimicrobial resistance genes (ARGs) (Robertson and Nash, 2018). The MOB-suite tool was evaluated in a benchmarking study, comparing various plasmid assembly tools (from Illumine sequencing data) and stood as the most efficient tool for predicting plasmids harboring ARGs in *E. coli* (Paganini et al., 2021). Additionally, compared to other platforms, MOB-suite has been shown to be the best in predicting plasmids contributing to the spread of ESBL genes (Paganini et al., 2021). The MOB suite results were consolidated using the platform Solu (Saratto et al., 2025), implementing default cut-offs of a minimum length of contigs of 1,000; minimum sequence identity for relaxases and replicons was 80%; and minimum coverage for replicons and relaxases was 80%. Any plasmid markers detected in chromosomal contigs (possibly indicating assembly errors) were excluded from reported results.

## 3 Results

### 3.1 Antimicrobial resistance phenotypes in *Escherichia* ***coli***

**FIGURE 1:**
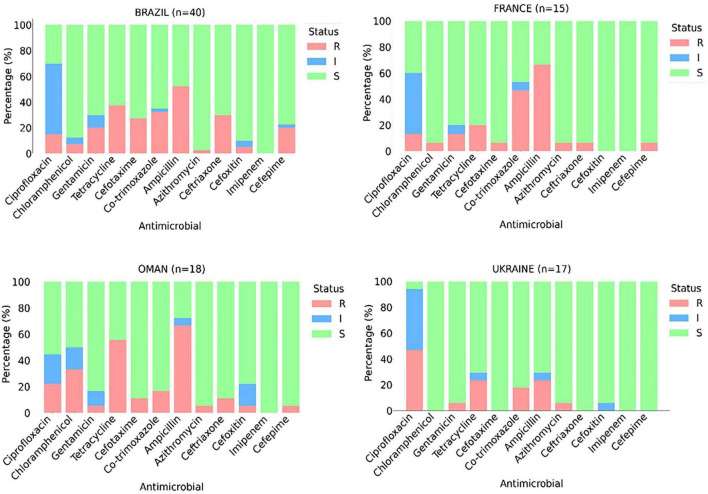
Phenotypic antimicrobial resistance (%) patterns of *Escherichia coli* isolates (*n* = 90) obtained from imported frozen broiler sampled from the United Arab Emirates market. The intermediate category (I, blue) represents the zone of inhibition between susceptible (S, green) and resistant (R, red).

Using enrichment culture procedures, *E. coli* were recovered from 248 out of 254 tested samples of frozen broiler carcasses. Figure 1 presents the phenotypic resistance profiles of 90 randomly selected *E. coli* from the carcasses originating from different batches from four different countries, of which 79 (87.8%) showed resistance to at least one agent and 62 (68.9%) were denoted as multidrug-resistant (MDR) (Figure 1). Overall isolates, the highest frequency of phenotypic resistance among the characterized *E. coli* isolates were against ampicillin (52.2%) and tetracycline (35.6%) (Figure 1). At the same time, none showed resistance to imipenem (Figure 1).

Country-specific analysis indicated substantial variation in resistance profiles (Figure 1). Among the 40 isolates from Brazil, 32 (80%) exhibited an MDR profile. A significantly higher frequency of resistance to cephalosporine (30% to ceftriaxone, 27.5% to cefotaxime, and 20% to cefepime) was evident among isolates from Brazilian chicken compared to other countries. Of the 18 isolates from Oman, 14 (77.7%) were classified as MDR. In addition, the isolates from Omani chicken carcasses had the highest rate of resistance to chloramphenicol (33.3%). The isolates from France and Ukraine exhibited comparatively lower resistance; 7 (46.7%) and 4 (23.5%) were categorized as MDR, respectively. On the other hand, fluoroquinolone resistance was highest in isolates from Ukraine, where ciprofloxacin resistance was observed in 47.1% of the characterized isolates.

### 3.2 Genotypic characterization and phylogenetic analysis

**FIGURE 2:**
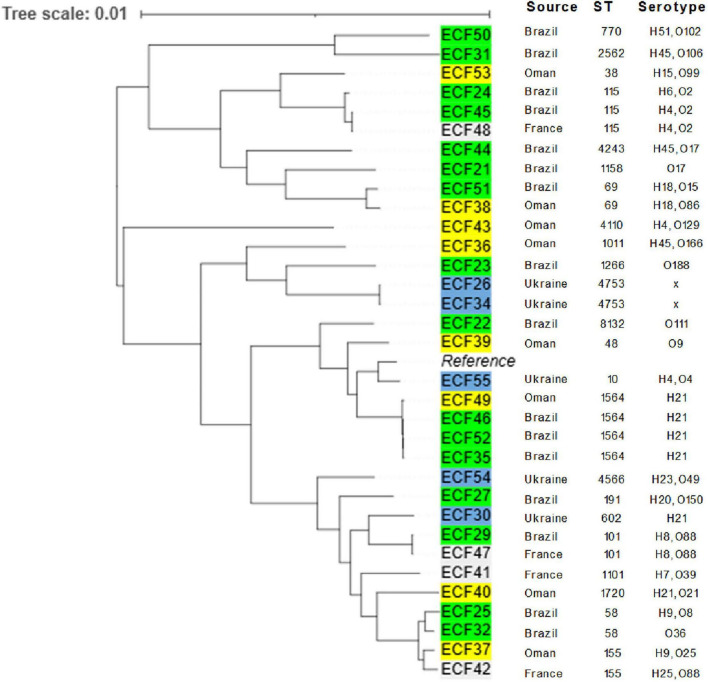
Midpoint rooted maximum likelihood phylogenetic tree of 33 whole-genome sequenced *Escherichia coli* isolates recovered from imported frozen broiler sampled from the United Arab Emirates market. The tree was rooted with *E. coli* strain K-12 substr. MG1655, assembly GCF_000005845.2 as a reference (Alignment length: 4,641,652 bp; phylogenetic tree statistics are provided in Supplementary Figure 1, and the SNP distances are available from Supplementary Spreadsheet Table 1.2).

Figure 2 presents a phylogenetic relatedness of 33 whole-genome sequenced *E. coli*. Phylogenetic tree statistics and alignment details are provided in Supplementary Figure 1. Isolates from Brazil and Oman showed close genetic relationships (Figure 2). However, no apparent phylogenetic clustering was observed based on country of origin, suggesting that resistant strains may be circulating through multiple poultry supply chains rather than confined to specific regions. The phylogenetic analysis revealed the identification of a diverse total of 22 distinct STs. The most frequently detected was ST1564, which was found in 4 isolates (12.1%) from two countries [Brazil (*n* = 3) and Oman (*n* = 1)]. One isolate (ECF55) was identified as *E. coli* ST10, which belongs to an international lineage of pathogenic (extraintestinal) *E. coli* of increasing clinical significance in humans. Pairwise single nucleotide polymorphism (SNP) distances among *E. coli* isolates are provided in Supplementary Table 1.2.

### 3.3 Identification of ARGs

Table 1 summarizes the resistance phenotypes and associated ARGs detected in the 33 whole-genome sequenced isolates. Notably, 19 isolates (57.6%) harbored more than five resistance genes, with eight isolates (24.2%) containing 10 or more. β-lactams-encoding genes were highly prevalent, with *bla*_*TEM*–1B_ gene, which confers resistance to penicillins, present in 12 isolates (36.3%) from all sources. The *bla*_*CTX*–*M*–8_ and *bla*_*CTX*–*M*–55_ were presented in 4 isolates (12.1%) (Table 1).

**TABLE 1:** Characterization of antimicrobial resistance profile of 33 whole-genome sequenced *Escherichia coli* isolates recovered from imported frozen broiler sampled from the United Arab Emirates market.

Country	Isolates	Antimicrobial resistance phenotype	Antimicrobial resistance genes
Brazil	ECF21	CIP;CTX;SXT;AMP;CRO;FEP	*aac(3)-VIa, bla*_CTX–M–2_ *,mdf(A),sul1, sul2*
	ECF22	CN;TE;CTX;SXT;AMP;CRO	*aac(3)-IV, aadA1, aph(3″)-Ib, aph(3′)-Ia, aph(4)-Ia, aph(6)-Id, bla*_CT–M–55_ *, mdf(A), tet(A), tet(B), sul1, sul2, dfrA1, fosA3*
	ECF23	SXT;AMP;CRO;FOX	*aadA2, mdf(A), sul1*
	ECF24	FOX	*mdf(A)*
	ECF25	TE;CTX;AMP;CRO;FEP	*aph(3″)-Ib, aph(6)-Id, bla*_CTX–M–55_ *, mdf(A), tet(B), fosA3*
	ECF27	CTX;AMP;CRO	*bla_*CT*–*M*–8_, bla*_CTX–M–55_ *, mdf(A), fosA3*
	ECF29	C;TE;AMP	*aadA1, aadA2, bla*_TEM–1B_ *, mdf(A), tet(A), sul3, fosA4, cmlA1*
	ECF31	CN;TE;CTX;SXT;AMP;CRO;FEP	*aac(3)-Via, aadA1, aadA2, bla*_CTX–M–2_ *, mdf(A), tet(A), sul1, sul2, sul3, qnrB19, dfrA12, cmlA1*
	ECF32	CN;CTX;SXT;AMP;AZM;CRO;FEP	*aac(3)-Via, aadA1, bla*_CTX–M–2_ *, mdf(A), sul1, sul2*
	ECF35	CIP;TE;CTX;AMP;CRO;FEP	*bla*_CTX–M–55_ *, mdf(A), tet(A), sul2*
	ECF44	CN;TE;AMP	*aac(3)-Via, aadA1, aadA2,bla*_TEM–1B_ *, mdf(A), tet(A), sul1, sul2, sul3, fosA4, cmlA1*
	ECF45	CIP;CTX;SXT;AMP;CRO	*aadA1, aph(3′)-Iia, bla*_CTX–M–8_ *, mdf(A), sul2*
	ECF46	TE;CTX;AMP;CRO;FEP	*bla*_CTX–M–8_ *, mdf(A), tet(A), sul2*
	ECF50	TE;SXT;AMP	*aadA1, bla*_TEM–1C_ *, mdf(A), tet(A), sul1, qnrB19, dfrA1*
	ECF51	C;TE;SXT;AMP	*aadA2, bla*_TEM–1B_ *, mdf(A), tet(A), qnrS1, dfrA8, floR, lnu(F)*
	ECF52	TE;CTX;AMP;CRO;FEP	*bla*_CTX–M–55_ *, mdf(A), tet(A), sul2*
France	ECF41		*mdf(A)*
	ECF42	SXT;AMP	*bla*_TEM–1B_ *, mdf(A), sul2, dfrA1*
	ECF47	C;TE;AMP	*aadA1, aadA2, bla*_TEM–1B_ *, mdf(A), tet(A), sul3, fosA4, cmlA1*
	ECF48	CIP;CTX;AMP;CRO;FEP	*aadA1, aph(3′)-Iia, bla*_CTX–M–8_ *, mdf(A), sul2*
Oman	ECF36	TE	*aph(3′)-Ia, mdf(A), tet(A), qnrS1*
	ECF37	CIP;TE;AMP	*bla*_TEM–1B_ *, mdf(A), tet(A)*
	ECF38	CIP;C;TE;CTX;SXT;AMP;CRO;FOX	*aadA1, aph(3′)-Ia, bla*_CMY–2_ *, bla*_TEM–1B_ *, mdf(A), mph(A), tet(A), tet(M), sul2, sul3, qnrS1, dfrA12, fosA4, cmlA1, lnu(F)*
	ECF39	C;TE;AMP	*aadA1, aadA2, bla*_TEM–1B_ *, mdf(A), tet(A), sul3, fosA4, cmlA1*
	ECF40	CIP;TE;CTX;SXT;AMP;CRO;FEP	*aadA1, aadA2, aph(3′)-Ia, bla*_TEM–1B_ *, mdf(A), tet(A), sul3, qnrS1, fosA4, cmlA1*
	ECF43		*mdf(A)*
	ECF49	C;TE;AMP	*aadA1, aadA2, bla*_TEM–1B_ *, mdf(A), tet(A), sul2, sul3, qnrS1, fosA4, cmlA1*
	ECF53	CIP;C;TE;SXT;AMP	*aadA1, aadA2, bla*_TEM–1B_ *, mdf(A), tet(A), sul1, sul3, dfrA1, fosA4, cmlA1*
Ukraine	ECF26	CIP	*mdf(A), sul2*
	ECF30	CIP;AMP	*bla*_TEM–1B_ *, mdf(A), fosA7*
	ECF34	CIP	*mdf(A), sul2*
	ECF54	CIP;TE;AMP	*bla*_TEM–106_ *, mdf(A), tet(B), sul2*
	ECF55	CIP;CN;TE;SXT;AMP	*mdf(A)*

AMP, Ampicillin; AZM, Azithromycin; C, Chloramphenicol; FOX, Cefoxitin; CTX, Cefotaxime; CRO, Ceftriaxone; FEP, Cefepime; CIP, Ciprofloxacin; CN, Gentamicin; TET, Tetracycline; SXT, Trimethoprim-sulfamethoxazole.

Resistance to aminoglycosides was commonly associated with the gene *aadA1* found in 14 (42.4%) isolates. Fluoroquinolone resistance gene *qnrS1* was present in 5 (15.2%) isolates, while tetracycline resistance gene *tetA* was identified in 17 (51.5%). Genes conferring resistance to sulfonamide *sul1*, *sul2*, and *sul3* were present in 8 (24.2%), 16 (48.5%), and 9 (27.3%) isolates, respectively (Table 1).

### 3.4 Plasmid diversity and predicted mobility

**FIGURE 3:**
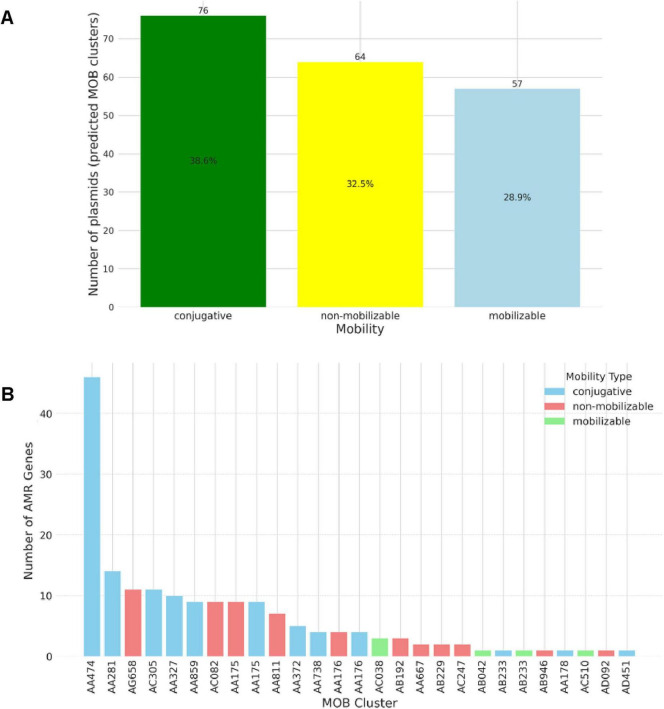
Predicted mobility of plasmids carried among 33 whole-genome sequenced *Escherichia coli* isolates recovered from imported frozen broiler sampled from the United Arab Emirates market. **(A)** Mobility is indicated by color: yellow, non-mobilizable; light blue, mobilizable; and green, conjugative. **(B)** Distribution of the number of AMR genes on complete plasmids MOB-suite clusters.

Isolate-specific plasmid details are provided in Supplementary File S3. Figure 3 presents the mobility distribution of plasmids in the 33 sequenced isolates, revealing that conjugative plasmids were the most prevalent, constituting 38.6% of the 197 predicted plasmids using MOB-suite analysis. Conjugative plasmids were detected in 21 isolates (63.6%), and the conjugative MOB-cluster AA474 stood as the most widely distributed among all clusters and harbored a sum of 47 putative antimicrobial resistance genes (Figure 3B).

**FIGURE 4:**
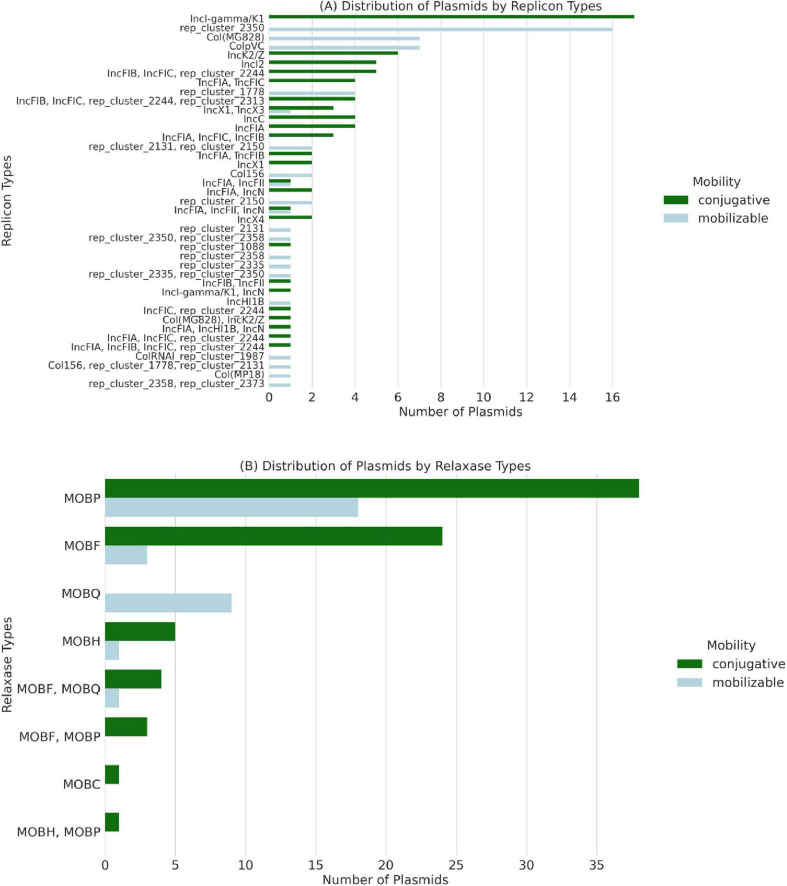
Classification of conjugative and mobilizable plasmids among 33 whole-genome sequenced *Escherichia coli* isolates recovered from imported frozen broiler sampled from the United Arab Emirates market. **(A)** The number of plasmids by replicon type is colored by their predicted mobility. **(B)** Number of plasmids by their encoded relaxases’ mobilization (MOB) type.

Further classification of plasmids by replicon type and mobilization potential, as shown in Figure 4, confirmed the conjugative nature of plasmids harboring ESBL genes. The IncI-gamma/K1 family was the most frequently detected in 18 isolates (54.5%) (Figure 4A). MOB classification of plasmids mobility is based on the concordance between their contents of the relaxase proteins. MOBP followed by MOBF were the most frequent relaxase types, with 37 and 24 plasmids containing these types, respectively (Figure 4B).

**FIGURE 5:**
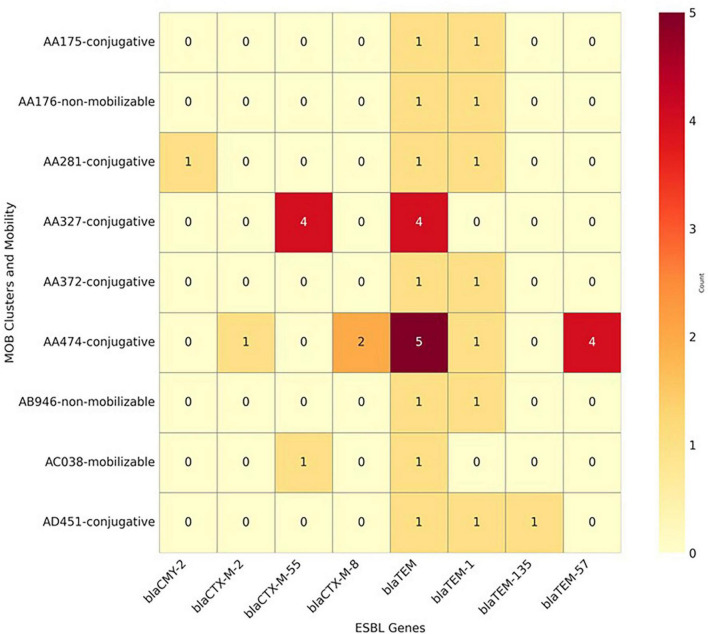
Combined heatmap showing the co-occurrence of β-lactam resistance genes, mobility genes, and MOB clusters identified in 33 whole-genome sequenced *Escherichia coli* isolates from imported frozen broilers sampled from retail markets in the United Arab Emirates.

Figure 5 presents the co-occurrence patterns among β-lactams-encoding genes, plasmid mobility types, and MOB clusters. The analysis identified that *bla*_CTX–M–55_ and *bla*_CTX–M–8_ primarily co-occurred with conjugative plasmids, increasing their potential for horizontal transfer. The *bla*_TEM–1B_ gene was detected on mobilizable plasmids in 4 isolates (12.1%), further contributing to the spread of resistance (Figure 5). The clustering pattern highlights specific MOB groups (e.g., AA474 and AA372) that carry multiple ESBL resistance genes, particularly within conjugative plasmids [MOB-cluster AA474 (IncI-gamma/K1, MOBP)].

## 4 Discussion

Scientific research indicates that imported foods can be potential carriers for the spread of AMR bacteria and associated genes (Warren et al., 2008; Harb et al., 2018). This study underscores the significant presence of antimicrobial-resistant *E. coli* in imported frozen broiler chicken retailed in the UAE, aligning with global concerns regarding foodborne AMR (Jung et al., 2022).

The phenotypic resistance patterns observed in this work reveal significant non-susceptibility to β-lactams, tetracyclines, and sulfonamides among *E. coli* isolates from imported poultry. This is an expected reflection of the traditional use of some of these antimicrobial classes in primary poultry production (Willis et al., 2023). However, tangible resistance rates to extended-spectrum cephalosporins were observed among *E. coli* characterized in the present work, especially among isolates from Brazilian chicken. This result is in concordance with previous reports on the elevated cephalosporin resistance in poultry-associated *E. coli* strains, attributed to the burdened use of 3rd generation cephalosporins at the farm level (Lentz et al., 2020; Hossain et al., 2022; Jung et al., 2022). Casella et al. (2018) in Brazil pointed to a high rate of extended-spectrum cephalosporins detection in *E. coli* from the meat and gut of chickens, with resistance genes found to be harbored on excessively diverse genetic elements (Casella et al., 2018). The risk assessment of antimicrobial resistance elements identified in imported foods warrants additional research.

Identifying *bla*_CTX–M–55_ and *bla*_CTX–M–8_ as the predominant genes conferring ESBL resistance in this study is noteworthy. The *bla*_CTX–M–8_ gene belongs to the CTX-M-1 family of β-lactamases and has been reported widely in South America, especially Brazil, in both clinical and food animal isolates (da Silva et al., 2022). The *bla*_CTX–M–55_ gene, a derivative of *bla*_CTX–M–15_, is among the most abundant ESBL genes internationally, particularly in Asia and the Middle East (Hadi et al., 2023). Studies indicate that *bla*_CTX–M–55_ has a higher catalytic efficiency than *bla*_CTX–M–15_, which confers stronger resistance to cefotaxime and ceftazidime (Yang et al., 2023), explaining the elevated rate of resistance to such antimicrobials among the isolates carrying such genes in the current study. As observed in this work, the concurrent detection of ESBL genes and fluoroquinolone resistance genes in *E. coli* isolates is particularly concerning because fluoroquinolones are widely used for treating serious Gram-negative infections (Wiener et al., 2016). Since 85% of the UAE’s chicken meat is imported, detecting MDR *E. coli* with multiple resistance genes highlights the diverse sources of antimicrobial resistance entering the UAE food supply.

The phylogenetic analysis in this study highlighted substantial genetic diversity within the sequenced *E. coli* isolates, with 22 distinct STs identified. The presence of ST1564 in isolates from Brazil and Oman suggests a possible common source or widespread dissemination of this lineage through international trade. The detection of *E. coli* ST58 in the present isolates is concerning, given that it is becoming an internationally reported uropathogen that could progress to sepsis (McKinnon et al., 2018). Along with colonizing humans, ST58 has been identified in poultry farm-associated environments (Benlabidi et al., 2023). Moreover, detecting ST10, even in one isolate (from Ukraine), is particularly significant as it is a recognized high-risk lineage associated with pathogenic (extraintestinal) *E. coli* infections in humans (Manges, 2016). Although ST10 and ST58 were found in a limited number of *E. coli* strains in this work, their detection in imported poultry reinforces the need for continued genomic surveillance to track the emergence and spread of these lineages of public health significance.

Our results show the vast plasmid diversity concerning their predicted clusters, replicon types, and genetic content. The high rate of predicted conjugative plasmids is concerning. Identifying conjugative plasmids carrying ESBL genes emphasizes the role of horizontal gene transmission in spreading AMR determinants (Neffe et al., 2022). The MOB-suite analysis revealed that conjugative plasmids were the most prevalent, with MOB cluster AA474 and the IncI-gamma/K1 plasmid family being the dominant replicon type. The conjugative nature of the MOB cluster AA474 (IncI-gamma/K1, MOBP) has been confirmed experimentally within multiple bacterial taxa (Castellanos et al., 2019). The high frequency of MOBP relaxases in the present study isolates suggests a potential risk of resistance gene transfer beyond *E. coli* to other bacterial species (Loyola Irizarry and Brito, 2023). IncI-gamma/K1 plasmids have been isolated from various epidemiologically non-related *E. coli* clones over different periods and considered as “epidemic” plasmid (Castellanos et al., 2019). Puangseree et al. (2022) found that the production of the ESBL phenotype is strongly linked (statistically associated) with IncI-gamma plasmid in *E. coli* from humans, pork, and pigs (Puangseree et al., 2022). This suggests ESBL genes may be localized on these plasmid replicon types, aligning with previous research in *E. coli*.

This study is one of the first in the Middle East to provide genomic insights into plasmid diversity, mobility, and relaxase distribution in foodborne *E. coli* strains. However, a notable limitation of this work is its reliance on Illumina short-read sequencing, which, while highly accurate for SNP analysis and resistance gene identification, does not allow for full plasmid reconstruction or complete resolution of complex mobile genetic elements (MGEs) (Paganini et al., 2021). Despite this limitation, this study provides valuable baseline data on AMR-associated plasmid families and relaxase types, enabling the identification of selected isolates of interest for further investigation. Future studies should utilize a hybrid sequencing approach by integrating long-read sequencing technologies to achieve complete plasmid assemblies, detect structural rearrangements, and elucidate the genetic architecture of multi-drug resistance plasmids (Sanderson et al., 2023). Such a hybrid approach (both short and long-read sequencing would enhance our ability to track the mobilization and persistence of ARGs in the food chain, ultimately strengthening surveillance and risk assessment strategies for antimicrobial resistance in imported poultry products.

Another limitation of this study is the relatively small proportion of *E. coli* isolates subjected to WGS. Only 33 isolates were sequenced, representing approximately 11.6% of all positive samples. However, this proportion is comparable to, or even exceeds, that used in other genomic surveillance studies ((Joensen et al., 2014; Habib et al., 2023b). In microbial genomics, sequencing subsets of 10–15% of isolates is generally considered sufficient for “initial” assessments of population structure, detection of dominant lineages, and antimicrobial resistance profiling (Joensen et al., 2014; EFSA Panel on Biological Hazards (Biohaz), 2021). From a statistical standpoint, the representativeness and diversity of the selected isolates are more critical than the absolute number. The 33 sequenced isolates were carefully selected to cover the variability observed across sources, locations, and phenotypic traits, ensuring meaningful insights into resistance gene distribution, sequence types, and phylogenetic relationships. Therefore, despite the limited WGS sample size, the study provides robust and informative genomic data on *E. coli* circulating in retail foods in the UAE.

## 5 Conclusion

Given the present study findings, there is a need for stringent regulations on the utilization of clinically important antimicrobials in poultry, particularly in some of the key exporting countries of poultry products. Implementing antimicrobial stewardship programs that promote responsible antibiotic use in primary animal production is essential to curb the spread of AMR. Strengthening surveillance programs that monitor antimicrobial resistance in imported food products can help detect resistant strains before they become widespread in the local market. By integrating genomic tools into routine surveillance programs, researchers and policymakers can better understand antimicrobial resistance dynamics and develop evidence-based strategies to manage the risks associated with resistant *E. coli* in food products. Additionally, increasing public awareness about the risks associated with antimicrobial resistance in foodborne bacteria is crucial for encouraging safer food handling practices and reducing the risk of transmission. This study specifically focused on the characterization of AMR determinants and plasmidome diversity among *E. coli* isolates, providing novel insights into imported poultry-associated strains in the UAE, while detailed virulence profiling remains an important subject for future research.

## Data Availability

All raw data of the genome sequencing are publicly available in the National Center for Biotechnology Information BioProject number PRJNA1219370 (https://www.ncbi.nlm.nih.gov/bioproject/1219370).

## Footnotes

1
https://platform.solu.bio/

2
https://www.ncbi.nlm.nih.gov/bioproject/1219370
