# Supplementary material for: Characterizing antimicrobial resistance and plasmidome diversity in Escherichia coli from imported frozen broiler chicken in the United Arab Emirates
Source: Front Microbiol. 2025 Jun 16;16:1590906. doi: 10.3389/fmicb.2025.1590906 (PMC12206764; doi:10.3389/fmicb.2025.1590906)
Supplement: Supplementary file 1 [file Table_1.docx]

**
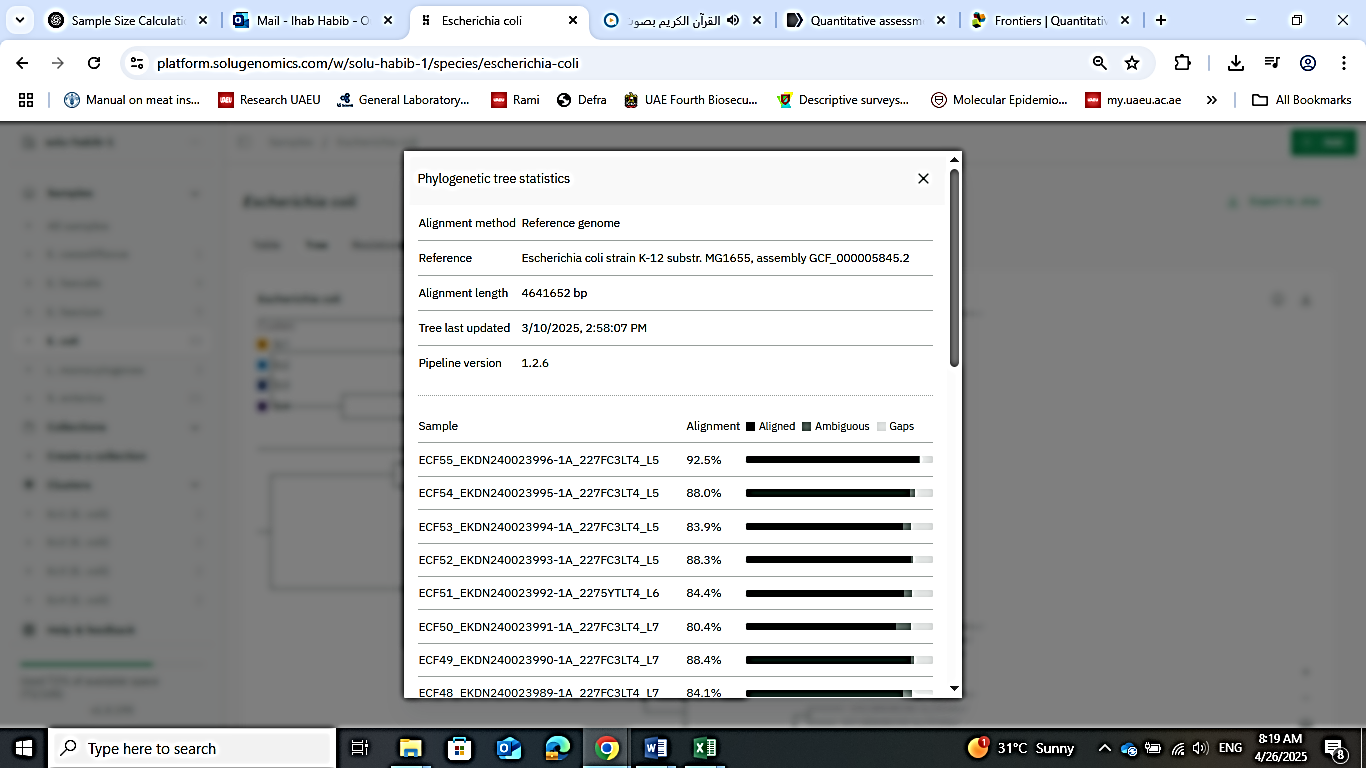
Supplementary Figure 1:**

**
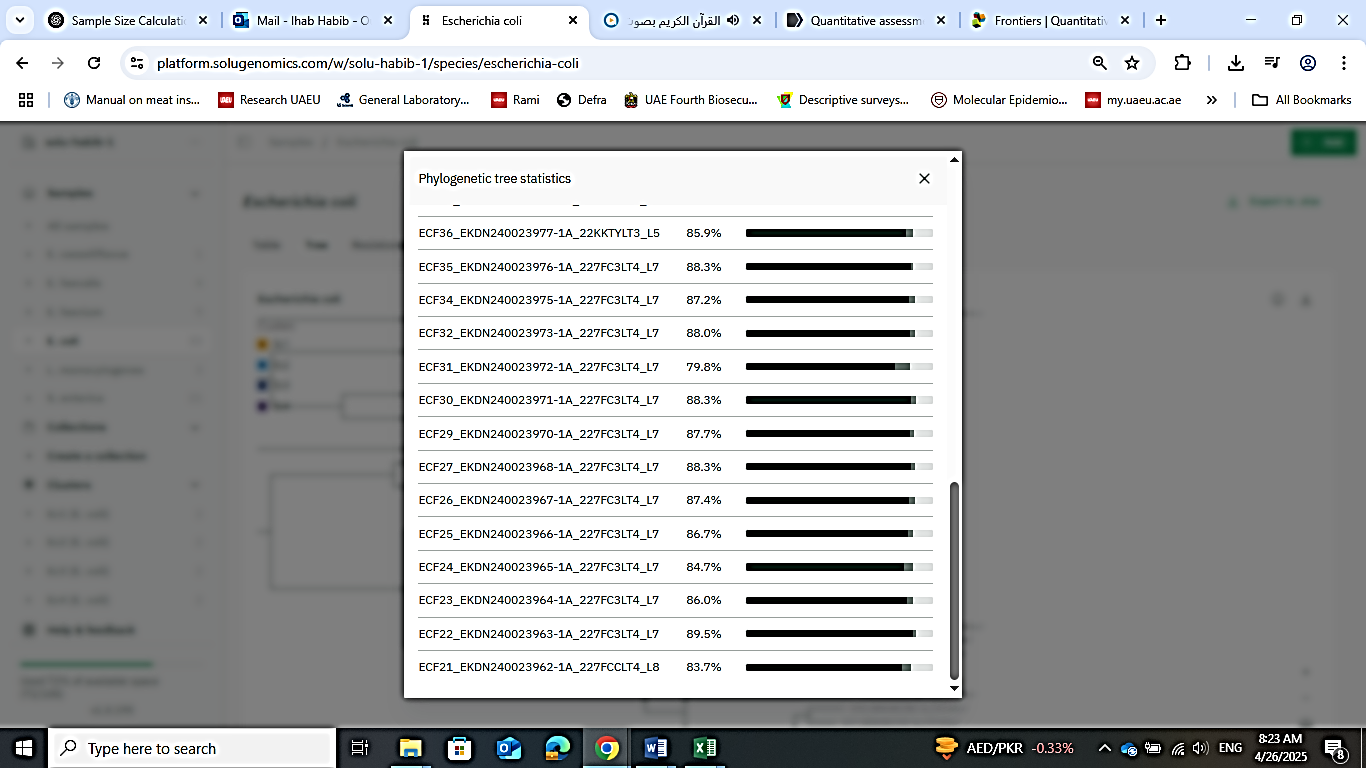

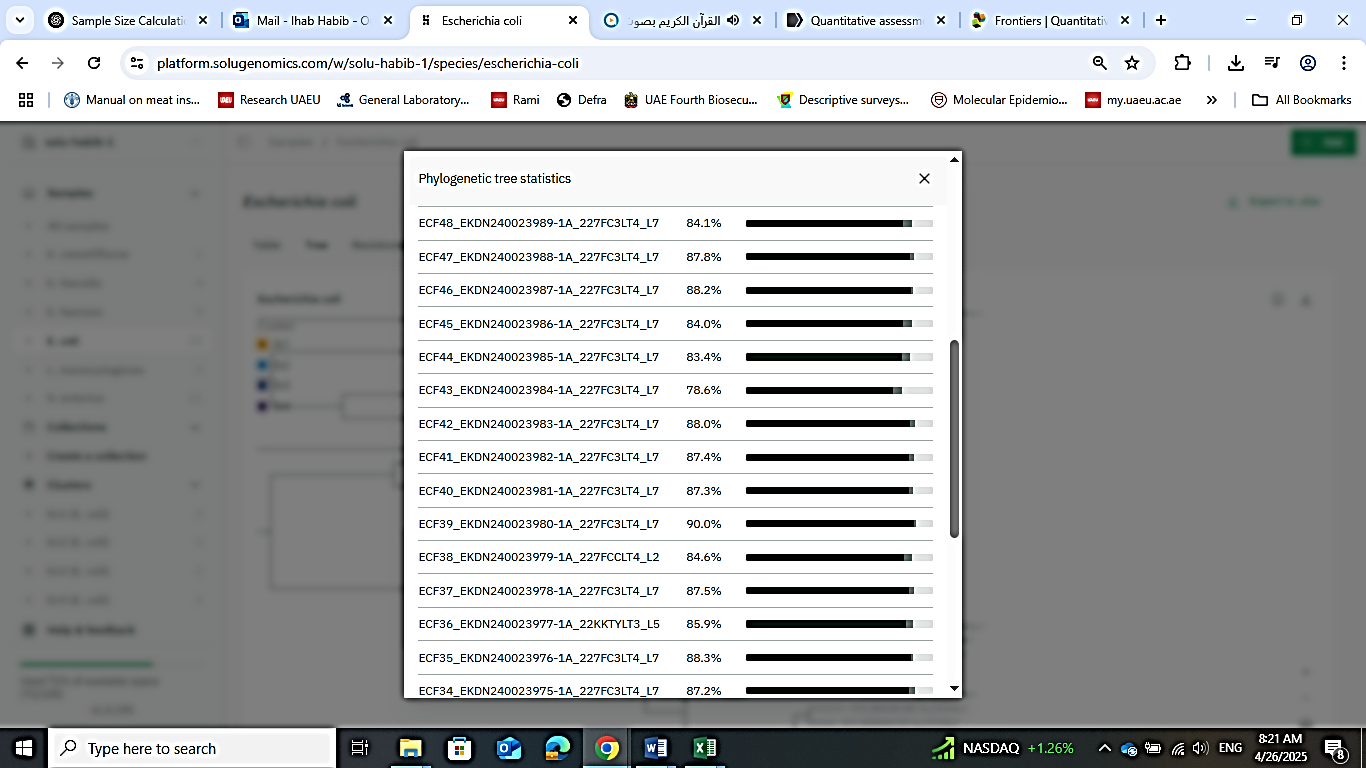
**SF.1. **Phylogenetic tree statistics** for the alignment of 33 whole-genome sequenced *E. coli* isolates from imported frozen carcasses sampled from UAE retails. The tree is midpoint-rooted with reference genome: *Escherichia coli* strain K-12 substr. MG1655, assembly GCF_000005845.2. Alignment length: 4641652 bp. The output in the figure below is a screenshot from output analysis by the online platform SOLU (Solu Healthcare, Inc., Finland, https://platform.solu.bio/
